# Motivation from Agency and Reward in Typical Development and Autism: Narrative Review of Behavioral and Neural Evidence

**DOI:** 10.3390/brainsci12101411

**Published:** 2022-10-20

**Authors:** Irene Valori, Laura Carnevali, Giulia Mantovani, Teresa Farroni

**Affiliations:** Department of Developmental Psychology and Socialisation, University of Padova, 35131 Padova, Italy

**Keywords:** agency, reward, ASD, autism, motor planning

## Abstract

Our ability to perform voluntary actions and make choices is shaped by the motivation from having control over the resulting effects (agency) and positive outcomes (reward). We offer an overview of distinct and common behavioral and neural signatures of agency and reward. We discuss their typical and atypical developmental trajectories, focusing on autism spectrum disorder (ASD), which is characterized by neurodiverse processes underlying action selection. We propose that reduced sensitivity to agency and reward in ASD may be related to atypical multisensory processes and motor planning, with potential for understanding restricted and repetitive behaviors. We emphasize the limitations of the existing literature, and prospects for future research. Understanding the neurocognitive processes that shape the way people with ASD select actions and perceive outcomes is essential to support not only learning, but also volition and self-determination.

## 1. Introduction

Our ability to perform actions and make choices is fundamental in our daily interactions with the world of physical and social objects. The link between a given action and its effects in the surrounding environment modifies our behavior and the underlying cognitive and neural processes, with meaningful effects on our acting, thinking, and learning. We can distinguish between the perception of control over the effects of one’s actions (agency) and the search for positive outcomes (reward). Understanding how these two different mechanisms give rise to a person’s actions and choices allows us to facilitate learning, and also volition and self-determination. This may be crucial for interventions that aim to support learning processes for people with neurodevelopmental conditions whereby agency and reward mechanisms can be affected.

## 2. The Role of Agency and Reward in Shaping Actions

In everyday life, we perform voluntary, goal-oriented actions for which we hold ourselves responsible. Agency can be defined as the perception of control over one’s own actions and the external world and can be traced back to the ability to recognize oneself as the cause of an event [1,2,3]. Before action execution, the motor system builds a prediction of its sensory consequences. The cortical connectivity between the frontal areas that plan voluntary movements and parietal areas that monitor outcomes is fundamental in retrospectively (i.e., after action execution) assessing the prediction–outcome match [2]. In case of alterations in the spatial and temporal contiguity between action and effect, the mismatch between predictions and actual outcomes would push the individual to search for an external cause of the event [4,5]. On the other hand, processes in the frontal cortex occurring before the initiation of action prospectively operate and underlie the subjective experience of one’s own voluntary action [2]. We sometimes consider ourselves authors of an event, even without being directly responsible for it. Whenever an event turns out to be in line with one’s intentions, there is a strong tendency to interpret it as self-generated [1]. For example, we push the crosswalk light buttons because we expect this to reduce the wait until the green light comes on. When the traffic light turns green, after variable and even prolonged time delays, we attribute this event to our action of pressing the button, even though there is no evidence of our role in turning the light green.

Agency arises from both implicit and explicit processes and can be distinguished into feeling of agency (FoA) and judgement of agency (JoA) [6]. FoA comes from implicit sensorimotor representation and can be considered the preconceptual component of agency. Altering the spatial or temporal contiguity between action and effect significantly disrupts this level of agency [7]. On the other hand, JoA consists of explicit recognition of oneself as the cause of an event. It is influenced by personal beliefs, contextual and social information [6], and is insensitive to factors of spatial and temporal contiguity between the action and its outcome [7]. Different measures have been employed in literature to capture explicit and implicit agency. Self-reported measures of explicit agency come from the person’s answer to the question, “Did you do that?” [2]. One of the most widely used implicit measures of agency is the intentional binding effect that refers to the tendency of agents to perceive the time interval between a voluntary action and a sensory stimulus as shorter than it actually is. More specifically, the onset of the voluntary action is reported later in time and awareness of the sensory feedback is temporally anticipated [8].

At the behavioral and motor level, the control-based response selection framework (CBRS) proposed that producing effects that are perceived as self-caused facilitates action selection and execution [9]. Indeed, the way we plan and control our movements is affected by several cognitive and sensorimotor features of actions [10]. We select response options more frequently and faster when they are associated with a higher probability of producing an effect, compared with no effect; thus, we are motivated and facilitated by having control [9]. At an implicit level, agency impacts motor parameters of actions (i.e., reducing reaction times), relies on action–effect temporal contingency (i.e., is disrupted by action–effect delays), and goes beyond individuals’ explicit judgements of agency [9]. In everyday life, it could be that fluently selecting an action makes it more likely that our intentions will be realized, and expected outcome achieved. Individuals reported greater perceptions of control over a given event when prime stimuli allowed for more fluent and immediate action selection [11]. According to this, habitual actions are accompanied by a strong sense of control and could therefore be sustained by agency mechanisms. Intriguingly, the motor system may be insensitive to abstract representations of the valence of an effect (i.e., receiving a positive or neutral effect does not change motor parameters of action) [12].

### 2.1. Neural Underpinnings

The neural signatures of agency involve several areas, such as the angular gyrus, temporo-parietal junction, supplementary and pre-supplementary motor areas, insula, dorsal medial-frontal cortex, and precuneus [13]. Importantly, explicit and implicit agency are differentiated at the neural level, with different areas being involved. Activation of the anterior insula seems to be particularly related to explicit positive agency (“that was me”), whereas the inferior parietal cortex is involved during externally driven action [14]. Several authors reported an increased activation of the angular gyrus in the inferior parietal lobe during explicit judgment of negative agency (“that was not me”) [15,16]. A recent meta-analysis of neural correlates of agency highlighted that subjective judgment of negative agency is associated with neural activity in the right superior temporal gyrus, left inferior parietal lobule, and left middle temporal gyrus, whereas no specific underpinnings of positive agency were found [17].

Implicit agency seems to specifically rely on the activation of the supplementary motor area (SMA) and pre-SMA [18,19,20,21], which are neural circuits responsible for the preparation, execution, and perceptual monitoring of voluntary actions [22]. Two neural signatures of implicit agency have been studied by previous literature, one relative to action preparation processes (readiness potential) and the other referring to the processing of action outcomes (N1 and P2). Indeed, intentional actions are preceded by a readiness potential (RP), a slow buildup of electric potentials associated with the neural activity involved in voluntary movement preparation [23]. Readiness potential involves early and late components: the early component is a negative ongoing activity that starts around 1 s before the voluntary movement; it is generated in the pre-SMA/SMA and related to the intention to move. The late component starts 500 ms before the voluntary movement and is thought to be a more specific motor preparation activity generated in M1 and premotor cortex. Importantly, RP is modulated by stimulus expectancy. For example, the expected sensory feedback in response to a voluntary movement influences RP amplitude and delays the latency of its onset [24]. In the pre-SMA, the readiness potential (RP) associated with the preparatory cortical activity that precedes voluntary actions is closely correlated with outcome binding [21]. These data support the involvement of SMA in predictive processes and suggest that premotor activity in fronto-parietal areas may play a crucial role in predictive processes and in the sense of agency.

The actual sensory feedback to an action is reflected by ERP components that have been associated with processing of action outcomes, such as auditory and visual N1 and P2. These components vary in timing and amplitude, in response to the stimulus generated by voluntary vs. externally generated actions [24,25]. In addition, some authors suggest an involvement of the cerebellum, posterior parietal cortex (PPC) [26], extrastriate body area (EBA), and superior temporal sulcus (pSTS) [27,28,29] in implicit aspects of agency. The cerebellum is involved in detecting the discrepancies between predicted and actual sensory consequences of a movement [30]. The PPC is a key area for monitoring the concordance between intended and visual consequences of self-produced actions. Finally, the EBA is active during self-generated movements [31].

Our actions are certainly shaped by the valence of their consequences. We prefer to perform actions associated with positive effects, which have a motivational value and can be defined as rewards. The neural substrates of reward [32] are distinguished from the ones devoted to agency, and have been long studied [33]. The ventro-medial prefrontal cortex (vmPFC) is involved in the representation of a stimulus value [34] and plays a critical role in encoding the expected outcome value of different actions. The medial orbito-frontal cortex (mOFC) is specialized in encoding the magnitude and value of positive and negative rewards and primary reinforcers [35], aids in decision-making processes based on cost–benefit gradients [36], and has a key role in forming associations between unconditioned stimuli and primary reinforcers [37]. The anterior cingulate cortex (ACC) is associated with reward anticipation [38] and in mediating cognitive control in uncertain contexts [39]. The ventral striatum (VS) acquires dopaminergic projections from the ventral tegmental area and is important in reward anticipation and processing of error feedback during learning [40], including social learning [41]. The nucleus accumbens (NAc) links reward to behavior, reward-related decision-making, and encoding motivational feedback [42]. Limbic structures, including the amygdala, that are critical for social cognition, particularly for face processing, are also involved when reward stimuli have a social nature [43].

Separate cortical processes are devoted to anticipating the reward value or its probability. Whereas reward value (e.g., gain or loss) is associated with a frontocentral P300 ERP component [44], reward probability is associated with a medial prefrontal ERP at similar latency [45]. Moreover, distinct brain mechanisms underlie reward anticipation and consumption. Though anticipatory mechanisms seem to be similarly involved for non-social and social rewards, the consumption of different types of positive outcomes may elicit different neural activities. Social rewards are mainly associated with amygdala activation, whereas monetary rewards are particularly associated with thalamus activity [46]. Other authors found that the magnitudes of both monetary and social rewards were related to the activation of the ventromedial prefrontal cortex and striatum [47].

**Figure 1:**
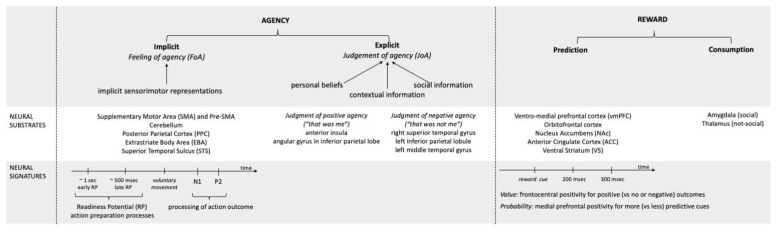
Schematic representation of the neural substrates and signatures of agency and reward.

In sum, research has extensively investigated agency and reward as separate mechanisms, showing that they contribute differently to action selection, shape distinct aspects of behavior, and emerge from distinct neural bases. Figure 1 summarizes the neural substrates and signatures that distinguish between agency and reward.

### 2.2. Bridging Mechanisms

Although the literature has separately investigated agency and reward, these mechanisms are closely interconnected during naturalistic interactions with the outside world. Indeed, when an action that the agent perceives as voluntary has a consequence that is interpreted as self-caused and positive, the two experiences are concomitant. For instance, people are biased in attributing positive outcomes to themselves [48], suggesting that the motivation derived from the sense of agency and from the positive valence of the outcome are indeed interconnected. Implicit (intentional binding) and explicit agency increases when people have a higher number of alternatives to select, they can make free (vs. instructed) choices, and the action outcome is positive [49,50]. Other researchers found that positive outcomes retrospectively enhanced implicit agency, which is particularly true when the outcome valence is unexpected or unpredictable [51].

Neural evidence suggests that agency and reward may act similarly in facilitating people’s selection of actions, specifically influencing the motor planning phase. Preparatory neural activity in motor and premotor areas anticipates voluntary movements and contributes to agency [21], which results in a sense of control that makes actions faster [9]. Similarly, there is evidence that monetary rewards make actions faster, with reward magnitude being associated with activation of pre-SMA and SMA brain areas, potentially promoting motor planning prior to action execution [52]. Reward signals have been also found in monkey dorsal premotor and primary motor neurons [53]. In addition, the reward system activation increases when individuals receive self-caused vs. random rewards [54], and can make proper choices instead of simple actions [55]. This evidence suggests that agency may modulate the way rewards are processed, thus fostering reinforced learning.

## 3. A Developmental Journey

The mechanisms underlying and associated with agency and reward are subject to specialization and tuning throughout child development and may undergo atypical trajectories under specific neurodevelopmental conditions. Decades of research demonstrated that infants learn through embodied sensorimotor contingencies, thus using their bodies to produce effects in the external world [56], with behavioral and neural markers of action–effect binding at around 3 months of age. After disrupting the action–effect contingency of infants’ movements, some of them showed EEG mismatch negativity and a reduction in their movement behavior, which respectively underpin violation of expectation and behavioral extinction, potentially related to reduced agency [57]. Moreover, infants at around 9 months of age are aware of the association between actions and effects, thus responding faster to events that they had previously actively produced than to action-independent events [58]. Other authors question the appropriateness of these methods for studying the sense of agency in preverbal children, and point out that the mere association between stimulus and response is not sufficient to constitute a minimal sense of agency, which should be distinguished from reinforced learning [59]. Additional studies have investigated implicit agency in school-aged children, who showed reduced temporal binding than adults [60,61]. Other authors found adult-levels of intentional binding in children from 6 years of age [62].

Notably, the threshold for detecting temporal biases between action and consequence may change during development. From the age of 4 to 15, there is a progressive decrease in the minimum temporal delay necessary for a person to be aware of the action-effect alteration [63]. Overall, the temporal interval within which multisensory stimuli are likely to be perceptually bound (namely, multisensory temporal binding window) gradually decreases up to adolescence [64]. The time window for intentional binding seems to be associated with manual dexterity, and is extended in children with developmental coordination disorder (DCD) [5]. In this population, reduced implicit agency was associated with depressive tendencies, thus contributing to the children’s well-being [5]. Contradictory findings come from adolescence, whereby researchers have found both reduced implicit agency compared with children and adults [65], and greater experience of implicit agency during mid-adolescence, which was mediated by a neural oversuppression of action outcomes (sensory attenuation) and over-reliance on motor preparation (late readiness potential) [25]. We can conclude that different sensitivities in detecting temporal biases could contribute to differences in implicit mechanisms of agency and impact the broader dimensions of child development and well-being.

As for the explicit judgment of agency, school-aged children and adults seem to be equally accurate in estimating their control over an event as a function of action–outcome congruency [48]. However, top-down processes, such as metacognition, change across the lifespan and affect children’s explicit agency up to later childhood. In particular, the outcome valence influences our causal attributions. A self-attribution bias that over-attributes positive outcomes to oneself and negative outcomes to external factors is pervasive in the general population but greater in children than adults [48]. For instance, children from 8 to 10 years old accurately judged a negative outcome as not self-caused, but believed they were responsible for positive outcomes that they did not actually cause [66]. Overall, school-aged children are happier when allowed to make choices among options, rather than being given only one option, thus being motivated by explicit agency [67]. However, in cases of a negative outcome, children’s emotions may worsen after self-determined choices compared with having no choice [67]. Moreover, children’s academic success is positively associated with their judgment of control, or explicit agency (i.e., believing that they know how to influence outcomes of success and failure in their academic life) [68]. Crucially, explicit agency is built on high-level cognitive processes (e.g., expectations, beliefs, and attitudes), which may be affected by some neurodevelopmental disorders. For instance, people with attention deficit and hyperactivity disorder (ADHD) show reduced self-attribution bias [69], which plays a fundamental role in their well-being [70]. Moreover, children with ADHD may be more sensitive to their action outcome valence, with an enhanced sensitivity to positive and negative outcomes and underlying atypicalities in neural reward circuits [71,72,73].

The nature of rewards may constitute a different degree of motivation depending on the context and the individual characteristics and age of the actor. Toddlers more frequently orient their attention toward social stimuli compared with non-social stimuli that respond to their gaze [74]. Later in childhood, monetary incentives may have stronger reinforcing value compared with social incentives when children perform cognitive tasks [75]. Finally, adolescence may be a critical period whereby social rewards are particularly valued [76]. However, different personality traits seem to mediate the extent to which a child benefits from different types of rewards, with higher reward-seeking tendencies and social skills being respectively related to higher benefits from monetary or social rewards [75].

In conclusion, both the feeling of control arising from agency and the positive valence of outcomes drive children’s actions. However, these mechanisms undergo developmental trajectories and specialize with age, potentially playing a role in atypical development.

## 4. Agency and Reward in Autism

The perception–action processes on which the sense of self is rooted are particularly affected by autism spectrum disorder (ASD). This neurodevelopmental condition is diagnosed from the very first years of a child’s life based on persistent and pervasive deficits in social communication and social interaction, as well as restricted and repetitive patterns of behaviors, interests, or activities [77]. Restrictive and repetitive behaviors may come along with atypical action selection processes, among which agency and reward play a crucial role. Understanding these mechanisms in ASD may shed light on how to promote learning, volition, and self-determination.

Using implicit measurements of agency, some researchers found differences in the autistic adult population. Participants were asked to press the spacebar whenever they wanted. Sensory feedback was presented after a variable temporal delay (i.e., 250, 450, or 650 ms), and participants were required to estimate the delay. Despite being accurate overall in their time perception, autistic adults showed reduced intentional binding compared with controls [78]. Another study on explicit agency in ASD showed that high-functioning autistic and neurotypical adults were equally able to judge whether a visual effect was self-caused or not [79]. Participants were asked to move a joystick and its cursor representation on a screen. The authors manipulated the degree of correspondence between participants’ actual movement and the visual feedback (i.e., the cursor movement). Half of the trials delivered synchronous visual feedback of participants’ real movement. The other half of the trials showed pre-recorded cursor movements from a randomly selected previous trial performed by the same participant. When analyzing explicit measures of agency by asking the question, “Did you perform the action on the monitor?” no significant differences emerged between the two groups [79]. These findings suggest a dissociation between explicit and implicit agency in people with ASD [80]. Although these considerations are based on very few studies and further investigation is needed, we can hypothesize that people with ASD experience a reduced sense of implicit agency, thus being less motivated by the sense of control that accompanies voluntary actions and self-caused events. To the best of our knowledge, there are no previous studies investigating agency in children with ASD, thus preventing us from understanding the developmental trajectory leading to any atypicalities we find in adult populations.

To understand agency in ASD despite the limited research on ASD populations, we can take a hint from studies on other clinical populations that have atypicalities in common with ASD. For instance, developmental coordination disorder (DCD) entails early-emerging persistent difficulties in the acquisition and execution of coordinated movements [77]. Motor coordination difficulties seem to be negatively associated with socio-affective abilities, thus being a potential bridge between DCD and ASD [81]. The sensory processes underlying explicit agency have been found to be different in children with DCD compared with neurotypical ones. Children were asked to make an action that would cause an effect after a random temporal delay, and to judge whether the effect was self-caused. The time window for agency was extended in children with DCD, negatively associated with manual dexterity and positively related to depressive symptoms [82]. As multisensory temporal binding windows are enlarged in ASD [83], this could also impact the emergence of implicit agency. Looking at the cognitive mechanisms of agency, some interesting insights come from attention deficit hyperactivity disorder (ADHD) research. The cognitive mechanisms underlying the inattentiveness and impulsive symptoms that characterize ADHD may also affect agency. For example, a self-attribution bias that over-attributes positive outcomes to oneself and negative outcomes to external factors is pervasive in the general population, but greater in children than adults, and reduced in ADHD [69]. However, no difference in self-attribution bias was found in ASD [84], suggesting that higher-order cognitive mechanisms of explicit agency may be unaffected.

Extensive literature has investigated the motivation from reward in people with ASD. Neuroimaging evidence showed that when anticipating monetary reward, NAc activity was reduced compared with neurotypical individuals, whereas when perceiving the actual reward, hyperactivation of VMPFC was observed, suggesting reduced motivation from rewards [37,85]. Additional evidence suggests that higher autistic traits are associated with enhanced neural activity related to reward anticipation, but do not modulate reward consumption [86]. Reduced motivation from rewards has been particularly found with respect to social rewards. Among children with ASD, researchers found reduced neural responses of VS to social rewards [87], an attenuated vmPFC response to a presentation of favorite faces [88], and reduced activation of frontostriatal networks during socially rewarded learning [89]. However, there was contradictory data on this, leaving it an open debate. Some studies have found decreased amygdala activation in children with ASD [85], whereas others have reported increased amygdala activation during social reward anticipation in adults with ASD [90]. These results suggest an atypical developmental trajectory in amygdala reactivity to social incentives [32]. Overall, the social motivation account of ASD that hypothesizes reduced motivation from social rewards seems to be supported by just over half of the studies in the literature [91], which leaves many open questions about individual differences and heterogeneity. Moreover, it is interesting to note that atypicalities in the reward system are also present in other neurodevelopmental conditions or psychopathologies, and may constitute a trans-diagnostic feature [92].

### 4.1. Underlying Mechanisms

The reduced sensitivity to implicit agency and reward that can be found in ASD may be related to atypical sensorimotor processes that underlie action–outcome binding, which would be pivotal for both agency and reward. People with ASD show broad differences at the multisensory level [93,94], with multisensory facilitation and higher reliance on unimodal processing [95], an extended (hence less precise and specialized) multisensory temporal binding window [83], and reduced integration of multimodal (e.g., audio-visual) cues [96]. They also experience atypical integration of interoceptive and exteroceptive stimuli [97], with delayed or reduced effects of visuo-tactile stimulation on proprioception during the Rubber Hand Illusion (RHI), resulting in less subjective feeling of ownership and self-location drift toward the rubber body [98,99,100,101].

Multisensory development goes hand-in-hand with motor development, in a perception–action cycle that allows the individual to learn from their actions [102]. From infancy, babies at increased likelihood for a later diagnosis of ASD manifest delayed and qualitatively different motor development [103]. Later in life, children with ASD show a variety of motor difficulties in the domains of praxis and fine and gross motor skills [104], with asymmetrical gait [105] and impaired postural stability [106]. Difficulties in underlying motor planning, monitoring, and prediction are frequently found in ASD [78,107]. At the neural level, children with ASD showed reduced event-related desynchronization before movements, which is interpreted as a sign of reduced motor preparation [108]. Altered movement-related potential in people with ASD may reflect abnormal activity of SMA during action planning [109]. Moreover, some authors reported altered dACC activity in ASD during response monitoring, which was correlated with repetitive behaviors [110] and social difficulties [111]. Ineffective motor planning seems to be associated with motor stereotypies [112], which are involuntary, restricted, and repetitive patterns of movements that limit the individual’s resources to learn and practice various purposeful actions [113,114]. Motor stereotypies are present in ASD, other neurodevelopmental conditions, and typical development [115]. Notably, motor-related cortical potentials in premotor areas, which anticipate voluntary motor actions, were found to be absent before stereotypy onset in typical development [112]. Beyond the mechanisms that distinguish agency and reward, these two processes underlying action selection share a mutual influence with motor planning processes. There is still no evidence in the literature that clarifies the link between multisensory and motor planning atypicalities and sensitivity to agency and reward in ASD. Trying to summarize the limited evidence from autism research and drawing on findings from other neurodevelopmental conditions that share similarities with ASD, we can hypothesize that the latter involves differences in low-level sensorimotor mechanisms, which are particularly fundamental to action–outcome binding and motor planning, and are pivotal for experiencing agency and reward. We speculate that reduced agency and reward sensitivity in ASD could have huge impacts on the way people learn and perceive their actions in the world. Individuals who are less motivated by the consequences of their choices may experience less opportunities for learning and self-determination.

A perspective that is becoming increasingly relevant to understanding how people with ASD learn from their action–outcome contingencies is that of computational neuropsychology, better known as the predictive brain or Bayesian account of ASD [116]. Every agent carries out an action on the base of prior knowledge about the context and expectations around action execution and its consequences (i.e., priors). Actual motor output and sensory effects are monitored during and after action execution to detect potential deviations from expectations (i.e., prediction errors). Though agency arises from minimal prediction errors, higher error rates make the agent revise their prior knowledge, thus promoting search for alternative explanations, and ultimately, promoting learning [117]. This predictive cycle takes on a central role in social exchanges, where partners’ predictions interact and influence one another. Sharing the same predictive model of the interaction facilitates interpersonal synchrony [118], defined as the temporal coordination of actions, emotions, thoughts, and neural and physiological processes [119]. ASD may entail atypical processes underlying the derivation of the most probable interpretations of the environment [120]. In different perceptual functioning, sensory inputs are weighted more than prior or contextual knowledge when building up perception of people with ASD [121]. Some behavioral and neurobiological evidence supports that they overestimate the volatility of the environment, at the expense of building stable expectations [122,123]. These predictive difficulties could be a common basis for the differences in agency and reward mechanisms found in ASD. Computational processes also play a crucial role in social exchanges, and can contribute to several neuropsychological conditions [124].

### 4.2. Intervention Perspectives

The investigation of the intra-individual and neuropsychological mechanisms that shape the way individuals with ASD select actions and make choices does not neglect that they are situated and emerge from social, cultural, educational, and political contexts that shape the contours of “ability” and “disability”. Removing barriers to volition and self-determination is crucial when offering support and learning opportunities to people with ASD. From early on in life, giving children with ASD a good degree of control over their social and non-social environment may have a great impact on their well-being and quality of life. For instance, multi-sensory environments (also called sensory or Snoezelen^®^ rooms) have been used to give children tools to control and modify their sensory environment. In such spaces, the child having control is a key element that mediates increased attention and reduced repetitive and stereotyped behaviors [125]. These findings suggest that providing control over sensory changes to children may create better conditions for learning.

The principle of “following the child’s lead” is also at the core of naturalistic developmental behavioral interventions (NDBI), in which the adult promotes social engagement and learning by following the child’s initiative and preferred activities [126]. According to these approaches, rather than using extrinsic artificial reinforcers to promote target behaviors, the focus is on leveraging each child’s preferred interests and stimuli to provide learning opportunities and broaden the child’s range of skills and interests. Although there may be reduced motivation from reward in people with ASD compared with neurotypical individuals [32,37,85], enhancing the positive valence of their actions’ outcomes is a well-established method to promote learning [126]. From this perspective, social rewards can be more effective than non-social rewards. When a child with ASD learns new words (e.g., says “train” for the first times while playing with a toy train), having an adult smiling, looking and pointing to the train and saying, “Yes, it’s a train!” facilitates learning more than a non-social reward (the train lighting up when the child correctly names it) [127].

In recent years, there has been mounting interest in the potential of applying digital and multimedia technologies for people with ASD. The idea is to design mixed realities, multimedia interactive activities, and immersive virtual environments aimed at fostering children’s sense of agency while exploring sensorimotor stimulation, cognitive training, and social exchanges in a fun and playful context that can be tailored to individual needs. Encouraging evidence has come from projects such as the European-funded MultiSensory Environment Design for an Interface between Autistic and Typical Expressiveness [128], the Magic Room: A Smart Space for Children with Neurodevelopmental Disorder [129], the Lands of Fog [130], and Nuieve Lab [131]. The potential of digital and immersive technologies lies in the degree to which they can be used to create ecological situations where it is very easy to control and manipulate sensory aspects and content for research and intervention purposes.

## 5. Future Research Perspectives

This literature review highlights areas that could be investigated in future research. First, the existing literature does not yet offer much information on how the neural underpinnings of agency and reward specialize during child development and how they may be involved in atypical trajectories. This is particularly true for the sense of agency, which is poorly studied across different age groups and clinical populations. The most popular tasks for studying implicit (i.e., intentional binding) and explicit agency (i.e., direct questions) are only usable with people who have good verbal skills and understanding of abstract concepts, thus limiting their appropriateness for young children and people with difficulties in verbal communication and abstract reasoning. Other recently proposed paradigms have been based on simple tasks of choosing between options and measuring the frequency of choices and kinematic parameters [12]. However, it remains to be clarified whether these indices are indeed representative of the sense of agency, and whether they can be used with populations other than neurotypical adults. Investigating the neurocognitive mechanisms underlying neurodiverse experiences of agency could be particularly relevant to understand several brain disorders [132].

Moreover, the extant literature mainly employed simple and un-naturalistic tasks that may have limited the capturing of the essence of agency, and its role in driving people’s actions and choices; agency should be further explored in ecological situations where the action–consequence link takes on real relevance for the person. In addition, the action–outcome properties that give rise to agency have been mainly studied using non-social outcomes. Agency is crucial during interpersonal exchanges, whereby each partner of the interaction influences the behavior of the other through his or her own verbal and non-verbal initiatives, and the feeling that they have an active role in the exchange [133]. It is well-known that social characteristics of stimuli involve different neural mechanisms from those devoted to processing non-social stimuli [134]. It would be important for future studies to examine the developmental trajectories of agency in social and non-social situations and shed light on potential distinctions between social and non-social agency. This becomes crucial for understanding interactions where the social aspects can be ambiguous, for example, during increasingly common virtual interactions, where people interact with either digital representations of humans, or with artificial intelligences. It has been suggested that if people believe they are virtually interacting with a real human being, the neural reward areas are activated (i.e., ventral striatum), whereas believing they are interacting with an artificial intelligence specifically activates attention areas [135]. It would be even more fascinating to leverage dual-brain neuroscience [136] to understand how two (or more) individuals share patterns of brain activity during reciprocal experiences of social agency and reward. This approach would have groundbreaking perspectives for the study of interactions between people with and without ASD. Indeed, it has recently been highlighted that reduced performance by autistic adults on standardized measures of social skills and motivation do not clearly correspond with their real-world social interaction outcomes [137]. Therefore, research should consider not only intra-individual autistic characteristics, but also inter-personal dynamics to understand whether people sharing similar experiences of the world may interact better with each other, regardless of the presence or absence ASD [138].

## Data Availability

Not applicable.
